# Implications of Mucin-Type *O*-Glycosylation in Alzheimer’s Disease

**DOI:** 10.3390/molecules30091895

**Published:** 2025-04-24

**Authors:** Nancy Vela Navarro, Gustavo De Nadai Mundim, Maré Cudic

**Affiliations:** Department of Chemistry and Biochemistry, Florida Atlantic University, 777 Glades Rd., Boca Raton, FL 33431, USA; nvela2016@fau.edu (N.V.N.); gmundim2020@fau.edu (G.D.N.M.)

**Keywords:** Alzheimer’s disease, amyloid plaques, neuroinflammation, proteolysis, MUC-type *O*-glycosylation

## Abstract

Alzheimer’s disease (AD) is one of the most common neurodegenerative disorders linked to aging. Major hallmarks of AD pathogenesis include amyloid-β peptide (Aβ) plaques, which are extracellular deposits originating from the processing of the amyloid precursor protein (APP), and neurofibrillary tangles (NFTs), which are intracellular aggregates of tau protein. Recent evidence indicates that disruptions in metal homeostasis and impaired immune recognition of these aggregates trigger neuroinflammation, ultimately driving disease progression. Therefore, a more comprehensive approach is needed to understand the underlying causes of the disease. Patients with AD present abnormal glycan profiles, and most known AD-related molecules are either modified with glycans or involved in glycan regulation. A deeper understanding of how *O*-glycosylation influences the balance between amyloid-beta peptide production and clearance, as well as microglia’s pro- and anti-inflammatory responses, is crucial for deciphering the early pathogenic events of AD. This review aims to provide a comprehensive summary of the extensive research conducted on the role of mucin-type *O*-glycosylation in the pathogenesis of AD, discussing its role in disease onset and immune recognition.

## 1. Introduction

### 1.1. Alzheimer’s Disease

Alzheimer’s disease (AD) accounts for 60–70% of dementia cases and is estimated to affect 27 million people worldwide and 6.9 million Americans, thus becoming the most common cause of dementia [1,2]. The projection is that by 2060, in the US, the number of patients who are 65 or older with AD will reach 13.8 million, posing a significant impact on the lives of affected individuals and their families and representing a financial challenge with an estimated national cost of USD 360 billion solely in 2024, while global yearly costs may exceed USD 1 trillion for patients within the AD continuum [2,3]. Since this disease mainly affects people older than 60 years, the increased lifespan translates into the exponential growth of AD cases [2,4], resulting in extensive attempts aimed at disease treatment. Despite strenuous research efforts, current treatments for AD fail to alter the progression of the disease and are limited to symptomatic management [5]. The two recently approved monoclonal antibody therapies show promise in slowing disease progression; however, they are associated with a risk of severe adverse effects [6,7]. Nonetheless, there are some exciting new leads in the development of new disease-modifying therapeutic strategies to combat the alarming rise of Alzheimer’s and other neurodegenerative diseases [8]. In this review, we will target specifically mucin-type *O*-glycosylation of proteins associated with the onset of AD, expanding on its contribution to disease development and immune recognition.

### 1.2. Hypotheses Surrounding Alzheimer’s Disease Onset

In 1907, Alois Alzheimer was presented at the asylum for mentally ill patients in Frankfurt am Main with a case of a 51-year-old demonstrating varying symptoms, including disorientation of time and space, change of personality, and rapid memory loss [9]. Considering that clinical observations were insufficient to classify this case as a recognized illness at the time, Alzheimer’s analyzed the patient’s post-mortem brain and its unique anatomical differences. The evenly atrophic brain was characterized by the loss of neurons, dense fibril bundles, and “miliary foci” dispersed in the cerebral cortex. Interestingly, modifications were also noted in the glial cells [9]. Although Alzheimer’s discovery of presenile dementia in 1910 led to the disease being named after him by his mentor, Emir Kraepelin [10], no other significant discoveries in the pathology of AD were made for several decades. Seeking to characterize proteins relating to the pathogenesis of the disease, Glenner and Wong [11] sequenced what is currently known as β-amyloid (Aβ), the protein responsible for Alzheimer’s “miliary foci”. The presence of amyloid plaques, multimeric aggregates of a polypeptide of about 40 residues, was identified as the core protein of AD [12]. Furthermore, the foundational work of Kidd [13,14] and Terry [15,16] in studying the brains of patients with AD led to the discovery of distinct paired helical filaments (PHFs), which give rise to dense neurofibrillary tangles (NFTs) and neuritic plaques. Further studies confirmed that the major constituent of such PHFs and NFTs is the microtubule-associated protein tau [17,18].

**Figure 1:**
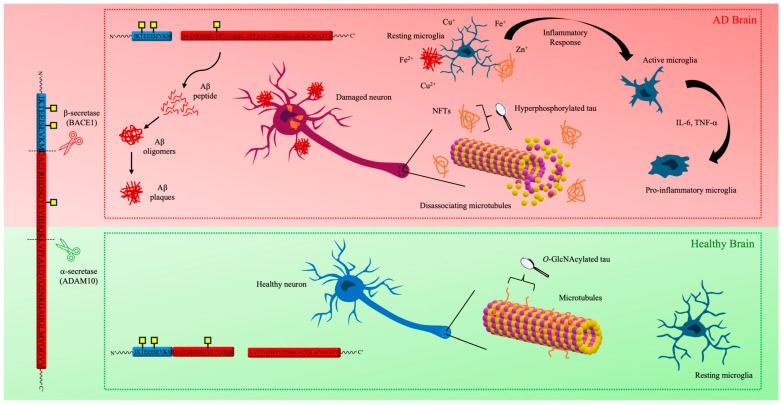
The sequence of major pathogenic events leading to AD (highlighted in red) proposed by the amyloid, tau, metal, and neuroinflammation cascade hypothesis.

Currently, it is well established that neuropathological hallmarks of AD consist of not only one but multiple microscopic features [19], including an abundance of amyloid plaques and NFTs, accompanied by microglial cell activation (Figure 1) [19,20].

#### 1.2.1. Tau Hypothesis

Studies related to the process of tubulin self-assembly into microtubules resulted in the discovery and classification of tau, a microtubule-associated protein [21,22,23]. The tau protein sequence can be divided into different regions, namely the microtubule-binding domain at the *C*-terminal and the projection domain at the *N*-terminal. While tau’s microtubule polymerization and stabilization property are associated with the 18-amino acid repeat of the C-terminal domain [24], the *N*-terminal’s composition of acidic amino acids followed by a proline-rich region may be responsible for its interaction with the plasma membrane and cytoskeleton [25]. Importantly, this protein has been found to undergo several different post-translational modifications (PTMs), including phosphorylation and glycosylation [26].

As mentioned, abnormal phosphorylation has been noted [27] under pathological conditions, while low levels of tau phosphorylation can be observed in healthy brains [28]. Specifically, 1977 marks the year of the discovery of tau phosphorylation [29], later linked to the downregulation of microtubule association. Further research demonstrated that PHFs and NFTs composition is enriched in hyperphosphorylated tau [28,29,30]. Considering its relevance in AD, tau phosphorylation became the most well-established of tau’s PTMs, with over 70 putative sites throughout the protein [31]. Therefore, the tau hypothesis highlights the effect NFTs carry in AD neurodegeneration, which is caused by this protein’s abnormal hyperphosphorylation and aggregation.

#### 1.2.2. The Amyloid Cascade Hypothesis

As postulated by Hardy and Higgins in 1992, the amyloid cascade hypothesis argues that the accumulation and aggregation of amyloid plaques in several different areas of the brain leads to neurodegeneration in AD [32]. The amyloid precursor protein (APP) is considered the primary source of Aβ peptides, which, when produced in excess or not properly cleared, form these plaques [12,33]. Proteolytic processing of APP generates two isoforms of Aβ, Aβ4040 and Aβ42. Although Aβ40 is more abundant in cerebrospinal fluid (CSF) [34,35], Aβ42 is the faster aggregating and fibril-forming isoform, given its increased hydrophobic nature through the presence of two extra residues at the C-terminus. Amyloid plaques found in the brains of patients with AD predominantly contain Aβ42, and the ratio of Aβ42 to Aβ40 in CSF is often used for AD diagnostic purposes [35].

Amyloid plaques are formed from aggregated Aβ species that can appear as monomers, dimers, oligomers, protofibrils, and fibrils [35,36]. In recent years, it has been proposed that Aβ oligomers present more neurotoxic character than mature fibrils or plaques, and given their early formation in the aggregation process, attention has been garnered on their specific roles in the progression of AD [37]. Interestingly, cognitive decline has been reported to begin prior to mature plaque buildup, during which elevated levels of oligomeric Aβ are observed in patients with AD [38]. While the exact structure of oligomers has not been fully elucidated, it is known that they differ in molecular weight. Oligomers are soluble and can disperse to other brain parts compared to fibrils [38]. Nevertheless, in pathological conditions, the clearance of either Aβ fibrils or oligomers is insufficient to offset the increase in the production of these peptides [39].

Thus, the question of whether plaques are the cause or a consequence of AD remains unresolved, and debate has intensified over the ability of the amyloid cascade hypothesis to explain the pathogenesis of AD [40,41,42,43]. Consequently, various aspects of this hypothesis have been investigated to broaden its scope. Currently, the amyloid hypothesis connects imbalanced production/clearance of Aβ and neurodegeneration in AD [44,45].

#### 1.2.3. Neuroinflammation Cascade

Emerging evidence reveals increased inflammatory markers in patients with AD, ultimately indicating that neuroinflammation has a key role in neurodegeneration, contributing to this process alongside plaques and NFTs [46]. Glial cells, such as microglia and astrocytes, have an essential role in maintaining homeostasis and providing immune responses [47]. Neuroinflammation results from the activation of these cells, characterized by the release of cytokines and chemokines [48]. Specifically, it has been found that Aβ plaques activate microglia [49,50], and these cells are also associated with NFTs [51], imposing a possible role in disease progression. Reactive microglia can be characterized as pro-inflammatory when actively engaged in the inflammatory response or anti-inflammatory after the resolution of the inflammation event, each releasing specific cytokines (e.g., IL-6 and IL-10, respectively) [52,53].

Importantly, C-type macrophage galactose lectin (MGL), known to be expressed by macrophages [54], was found to be induced in anti-inflammatory microglia [55]. This receptor recognizes glycan structures in a Ca^2+^-dependent manner and plays an important role in immune response [54]. Specifically, it preferentially binds to *O*-linked *N*-acetylglucosamine (GalNAc) [56], the carbohydrate that initiates complex glycan chains, seen in mucin proteins; thus, it is categorized as mucin-type *O*-glycosylation [57]. Considering the increased expression of this glycosylation in AD [58] and its potential to modulate the immune response, questions arise about the role mucin-type *O*-glycosylation plays in the neuroinflammatory cascade.

#### 1.2.4. Metal Hypothesis

In 2008, Ashley I. Bush and Rudolph E. Tanzi proposed the “Metal Hypothesis of AD”, stipulating that Aβ-metal interactions promote the neuropathogenic aspect of Aβ in AD [59]. Since then, essential metals, such as iron, zinc, and copper, have been regarded as potential critical players in the pathogenesis of AD. Considering that their homeostasis is altered in affected patients, in addition to heavy metal concentration, extensive research has tried to further elucidate their role in the development of the disease [60]. Interestingly, metals have been found to contribute to the AD cascades, representing a key player in tau hyperphosphorylation, Aβ aggregation, and neuroinflammation [60]. Therapeutic interventions have predominantly targeted the inhibition of metal-mediated interactions, primarily through the use of chelating agents capable of sequestering metal ions and disrupting the pathological Aβ-metal ion coordination [61]. Regardless, continued research is essential to advance our understanding of the multifaceted pathophysiological processes involved in this aspect of the disease.

Recent evidence indicates that patients with AD exhibit abnormal glycan profiles [62,63,64,65] and that many AD-associated molecules—beyond the key proteins APP and tau—are either glycosylated or involved in glycan regulation [66,67]. Furthermore, cross-talk between *N*- and *O*-glycosylation pathways in AD has been observed, involving alterations in both pathways that impact protein function and may contribute to disease pathogenesis [68]. Understanding the role of *O*-glycosylation is, therefore, essential for unraveling early pathogenic mechanisms. Glycosylation may serve as a unifying factor, linking various hypotheses on Alzheimer’s disease progression.

## 2. Post-Translational Modification in Alzheimer’s Disease

Eukaryotic cells rely on protein PTMs as a vital process to enhance and diversify protein functions beyond the genetic code’s instructions [69,70,71]. As PTMs are involved in regulating almost all cellular events and maintaining cellular homeostasis, the malignant transformation of primary cells or cellular neurodegeneration is usually accompanied by changes in protein’s PTMs [72,73,74,75].

### 2.1. Methylation

Methylation is dynamically regulated by methyltransferases and methylases, enzymes responsible for the attachment and removal of methyl groups to either lysine or arginine residues. While the latter has been reported to be mono-, symmetric di-, and asymmetric di-methylated, lysine can be mono-, di, and tri-methylated [76]. Several methylation sites have been identified in tau, including in the *N*-terminal region and the microtubule-binding domain [77], and have been implicated in enhancing tau aggregation in AD [78]. Furthermore, this PTM is closely associated with epigenetic regulation, particularly through DNA and histone methylation, which may impair neuronal function and contribute to the progression of the disease [79].

### 2.2. Ubiquitylation

Ubiquitylation plays a crucial role in the proteasomal degradation of misfolded proteins and aggregated peptides, ensuring cellular protein homeostasis. Several proteins implicated in AD, APP, and tau exhibit abnormal patterns of ubiquitylation. Impairment of this regulatory mechanism by Aβ peptides leads to the accumulation of neurotoxic aggregates, thereby contributing to AD pathogenesis [80]. Notably, elevated levels of ubiquitylated tau have been detected in PHFs and CSFs of individuals with AD. Despite the extensive ubiquitylation, PHF-tau evades proteasomal degradation and instead accumulates within neurofibrillary tangles (NFTs), which is characteristic of AD pathology. The precise molecular mechanisms underlying this resistance to degradation, however, remain to be elucidated [80].

### 2.3. Acetylation

The attachment of acetyl groups to lysine residues, also regarded as acetylation, regulates several cellular functions [81]. Although minimal APP acetylation is reported in the literature [82], tau can be acetylated at several different sites [83]. Studies indicate that tau acetylation can facilitate microtubule dissociation due to neutralization of the positive charge, induce tau aggregation into oligomers and short fibrils, and disturb tau degradation [80]. Notably, acetylation and ubiquitination target overlapping lysine residues, and acetylation also shares modification sites such as Lys163 and Lys174 with methylation. This convergence allows these PTMs to competitively modify the same amino acid residues, potentially resulting in distinct and context-dependent functional outcomes [80].

### 2.4. Phosphorylation

Phosphorylation is defined as the addition of a phosphate group that can occur at threonine, tyrosine, or serine, the latter representing the most abundant option [84]. Importantly, the reversible addition and removal of the negatively charged group through the action of kinases and phosphatases, respectively, have a great implication for a protein’s conformation and activity [84] and ultimately may lead to various diseases, including neurodegeneration [85,86]. The precursor protein for Aβ peptides, APP, has been identified with several possible phosphorylation sites, and they have been closely linked with AD progression [87]. Tau hyperphosphorylation is one of the most well-known examples of PTMs associated with AD [27]. It is worth noting the complex relationship between tau phosphorylation and neurodegeneration. Although the addition of phosphate groups at some positions has been shown to promote tau-microtubule dissociation, the same modification can at the same time decrease tau aggregation susceptibility [88]. Nonetheless, targeting phosphorylated tau is a promising approach for developing therapies to slow down tau hyperphosphorylation and aggregation in AD.

### 2.5. N-Glycosylation

**Figure 2:**
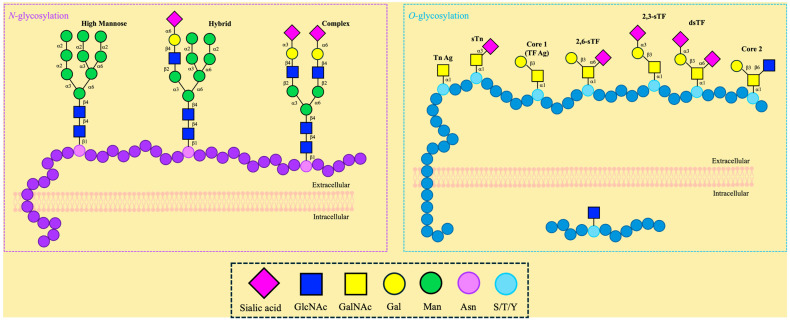
Different types of *N*- and *O*-glycosylation.

The covalent attachment of glycans to the side chain of asparagine in the endoplasmic reticulum is referred to as *N*-glycosylation, following the N-X-S/T (X ≠ P) consensus sequence [89]. All eukaryotic *N*-glycans share the common core sequence Manα1,3(Manα1,6)Manβ1,4GlcNAcβ1,4GlcNAcβ1-Asn-X-Ser/Thr, and further processing determines the oligosaccharide classification as oligomannose or high-mannose, complex, or hybrid (Figure 2). The attachment of unsubstituted terminal mannose glycans extending the core characterizes the oligomannose, whereas GlcNAc (*N*-acetylglucosamine)-initiated “antennae” are determined to be complex, having the possibility to be further extended by lactosamine (GlcNAcβ1,4Gal) units, often capped by sialic acid; hybrid, as depicted in the name, accounts for the mannose extension from the Manα1,6 arm of the core and one or two antennae initiated by the addition of GlcNAc in the Manα1,3 arm [90]. As a result of these oligosaccharide additions, protein folding, stability, and function are affected [91]. APP has been reported to contain this PTM at asparagine 467 and 496 [92]. Additionally, abnormal tau *N*-glycosylation in patients with AD has been reported [64,93,94]. Consequently, this PTM demonstrates a correlation with AD [95]; however, it is beyond the scope of this review.

### 2.6. O-Glycosylation

If glycans are attached to the hydroxyl group in the side chains of serine, threonine, or, less commonly, tyrosine, then it is classified as *O*-glycosylation (Figure 2). The attachment of monosaccharides is carried out by glycosyltransferases, and it can be initiated by several different carbohydrates, such as GlcNAc and GalNAc [96,97]. Contrary to *N*-linked, *O*-linked glycosylation does not follow a single consensus sequence, preventing accurate predictions and the discovery of novel glycosylation sites [98].

*O*-glycosylation can be differentiated into two major groups characterized by their compartmentalization, namely (i) nucleocytoplasmic and (ii) extracellular *O*-glycosylation (Figure 2). Alongside location, these categorizations also differentiate the possible glycan attachment. Only single *O*-GlcNAc moieties have been identified in residues of nuclear and cytoplasmic proteins; conversely, extracellular *O*-GalNAc-containing glycans are present in secreted or cell surface proteins [99].

Approximately 40 years ago, Torres and Hart discovered the novel β-*O*-linked GlcNAc [100]. Other studies confirmed the presence of this glycan on tau, and, interestingly, *O*-GlcNAcylation has been reported to be closely related to regulating tau phosphorylation [101,102]. In patients with AD, downregulation of glucose metabolism leads to decreased *O*-GlcNAcylation of tau, ultimately resulting in tau phosphorylation at the AD-related sites, suggesting this glycan has a neuroprotective role [103]. In addition to tau, APP has also been identified to contain *O*-GlcNAc [104], and its relevance in trafficking and processing has been assessed by multiple research groups, demonstrating that this PTM can attenuate Aβ production [105,106].

*O*-GalNAc-containing glycans are known as “mucin-type *O*-glycans” and are primarily found on mucins, a family of high molecular weight, highly glycosylated glycoproteins that exist as secreted and membrane-associated glycoproteins [80,81]. Mucin glycosylation is initiated by the covalent attachment of GalNAc to, usually, serine or threonine residues [107,108]. Two unique features of this type of glycosylation include the fact that this reaction takes place in the Golgi apparatus, succeeding protein folding, and that a large homologous polypeptide GalNAc-transferase family catalyzes the first step, which is the GalNAcα1-*O*-Ser/Thr linkage [109]. Glycosyltransferases supplement additional sugar moieties to extend the complexity and introduce a variety of eight main core structures [108,110,111]. The simple GalNAc, also called the Tn antigen, is composed of GalNAcα1-*O*-Ser/Thr, and the sialylated antigen sTn is formed by the addition of the *N*-acetylneuraminic acid (NeuAc) through an α2,6 linkage, thus forming Neu5Acα2,6-GalNAcα-*O*-Ser/Thr. Galactose (Gal) may extend the Tn antigen through a β1,3-linkage instead, thus forming the TF antigen, also known as T antigen or core 1 (Galβ1,3-GalNAcα-*O*-Ser/Thr), which can be sialylated at two different positions: Neu5Acα2,6- and Neu5Acα2,3-Galβ1,3-GalNAcα-*O*-Ser/Thr (2,6-sTF, 2,3-sTF). The extension of the Tn antigen with GlcNAc through a β1,3-linkage forms core 3 (GlcNAcβ1,3-GalNAcα-*O*-Ser/Thr). The addition of a β1,6-linked GlcNAc to cores 1 and 3 results in the formation of cores 2 and 4, respectively. These cores can be further elaborated by GlcNAc, Gal, fucose, and sialic acid [107]. This type of *O*-glycosylation has been reported in APP [58,112,113,114,115,116] and is recognized for its effect on processing fate, Aβ aggregation, and immune response modulation—topics that will be explored further in this review [117,118,119].

## 3. Implications of APP Mucin-Type *O*-Glycosylation in APP Processing

One of the widely accepted hypotheses for the onset of AD surrounds the continuous accumulation of Aβ peptides in the extracellular neuronal space until fibrillar plaques are formed, leading to neurodegeneration. Aβ peptides are a proteolytic fragment of APP, a type I transmembrane protein found in neurons and various epithelial cells. APP has been linked to synaptic functioning and signaling [120], and the focus of most of the research conducted on APP has revolved around its proteolytic processing in AD, either through the non-amyloidogenic or amyloidogenic pathways (Figure 1) [39,117,121,122]. In recent years, the discovery of glycosylation sites near the enzyme cleavage sites has warranted the study of the role of glycosylation in the processing of APP and consequent Aβ plaque formation. Therefore, the structure of APP, its proteolytic processing, and the effect of glycosylation on its neurotoxic fate will be explored below.

### 3.1. APP Structure and Physiological Functions

**Figure 3:**
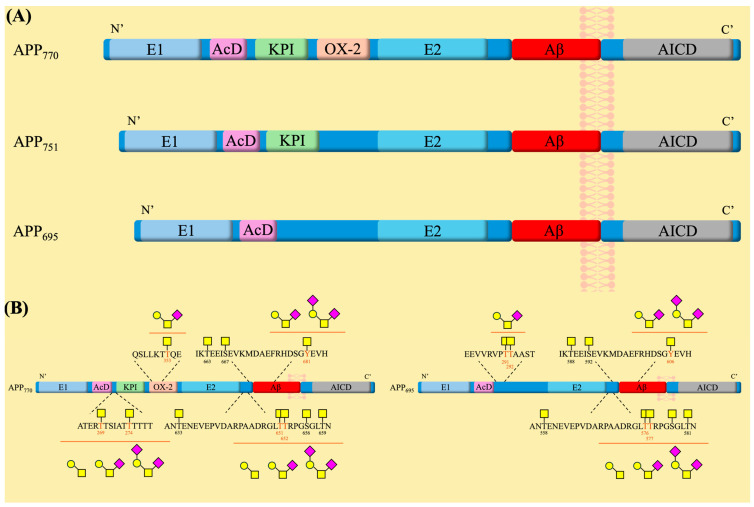
(**A**) The amyloid precursor protein family represented by 3 isoforms and highlighting the main sequence domains within each family member. (**B**) The glycosylation sites are present on the APP770 and APP695 isoforms. Symbol nomenclature for glycans: a yellow square for *N*-acetylgalactosamine (GalNAc), a yellow circle for galactose (Gal), and a pink diamond for sialic acid.

APP belongs to a family that includes itself and two other APP-like proteins (APLPs), which are characterized by a long extracellular *N*-terminal domain and a smaller disordered cytosolic *C*-terminal region [120]. While they all share conserved sections in their ectodomains, it is only APP that contains the transmembrane-bound Aβ sequence [120]. APP exists in different isoforms arising from variations in the splicing of exons 7 and 8 during DNA transcription, with three isoforms being the most common—APP695, APP751, and APP770 [123]. All three isoforms contain two distinct extracellular domains, E1 and E2, which are connected by a flexible acidic domain (AcD) and an APP intracellular domain (AICD) (Figure 3) [123,124]. The E1 and E2 domains contain the copper-binding and heparin-binding sites and, consequently, play a role in the regulation of neuronal copper homeostasis and cell adhesion interactions with extracellular proteins, respectively [123]. On the other hand, not much is known about the functions of the AcD and AICD domains. It has been suggested that the AcD acts as a flexible linker between the two larger domains [124], and AICD participates in interactions with apoptosis-related proteins [125]. The APP751 and APP770 isoforms possess an additional Kunitz-type serine protease inhibitor (KPI) domain located between E1 and E2. The APP770 isoform also includes an OX-2 sequence, a 19-amino acid sequence similar to a region of the OX-2 antigen [123]. These two domains have been reported to influence protein-protein interactions in peripheral cells [120]. Notably, both the KPI and the OX-2 regions are missing on APP695, which is the major isoform found in neuronal cells [123], suggesting a more specialized role for it. In the brain, APP695 has been linked to and is believed to have a role in regulating neurogenesis, synapse plasticity and maintenance, cell adhesion, neurotransmission, and memory development [120]. Overexpression of APP in mice models was shown to lead to neurodegeneration, while the knockdown of APP was shown to decrease cognitive functions and render the brain more susceptible to mass loss [126]. Consequently, APP has been extensively studied for its involvement in AD pathogenesis [123,127,128,129,130].

**Figure 4:**
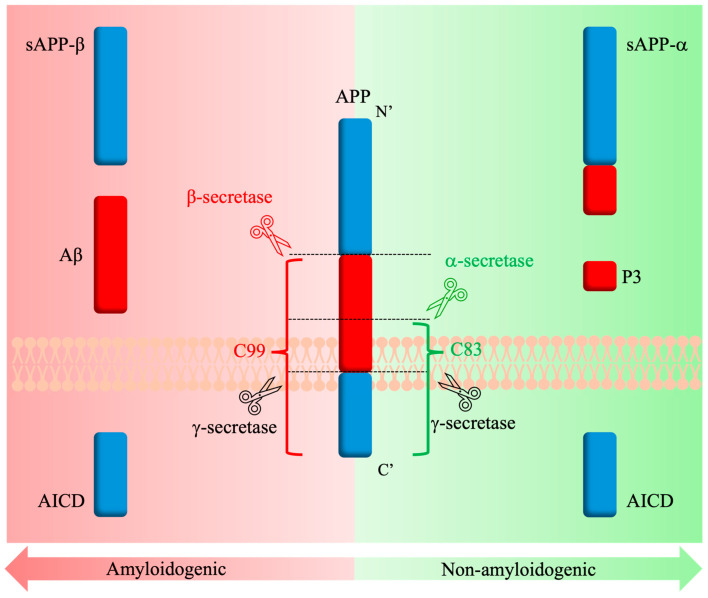
Amyloidogenic and non-amyloidogenic APP processing pathways in AD.

The processing of APP is a stepwise occurrence that is mediated by several different proteases and secretases [121]. It is widely accepted that APP undergoes two possible cleavage pathways in AD: the amyloidogenic and non-amyloidogenic pathways (Figure 4). In the case of the non-amyloidogenic pathway, α-secretase activity was originally attributed to various members of the ADAM (a disintegrin and metalloproteinase) family [131]. The presence of ADAM10 in the brain and the increased processing of APP upon its overexpression led to the conclusion that ADAM10 is the main α-secretase involved in non-amyloid processing [132]. The pathway begins with APP cleavage by ADAM10 within the Aβ region (K687~L688), releasing the soluble *N*-terminal domain fragment (sAPPα) into the extracellular space and further promoting the non-amyloidogenic pathway by its inhibitory interactions with the β-secretase [127]. The remaining membrane-bound C83 fragment undergoes cleavage by γ-secretase, resulting in the release of AICD and the P3 fragments [127].

Similarly, in the case of the amyloidogenic pathway, it was originally believed that two different enzymes from the BACE (β-site APP-cleaving enzyme) family are involved in the processing of APP. However, it was later confirmed that BACE1 makes up most of the β-secretase content in the brain, and its overexpression increased Aβ peptide formation [133,134]. Furthermore, the Swedish mutation (K670N/M671L) and the recessive mutation (A673V) notably enhance APP as a substrate for BACE1 and facilitate its amyloidogenic cleavage [114,128]. The BACE1 pathway processing begins with the cleavage of APP by BACE1, producing a soluble extracellularly released (sAPPβ) fragment and a membrane-bound C99 domain [127], much like how the non-amyloidogenic pathway begins. Interestingly, the relatively small changes in sequence between sAPPα and sAPPβ result in differences between their biological effects upon release. For example, sAPPβ is described as a ligand to signal neuronal cell death via caspases [135]. The remaining C99 membrane-bound fragment has been reported as neurotoxic and promoting neuron degeneration [121]. As with the non-amyloidogenic pathway, cleavage by γ-secretase follows, and the Aβ peptide is released, along with the AICD fragment, which then serves as a regulator for the trafficking of intracellular APP [127]. Differences in γ-secretase cleavage sites lead to the release of two major forms of Aβ, Aβ40 and Aβ42 [122]. Studies have shown that the accumulation of these Aβ peptides on neurons leads to cytotoxicity, cell loss, and neuroinflammation [127,136,137].

In recent years, the research surrounding the processing of APP has expanded to include several other enzymes that can influence the progression of AD. Matrix metalloproteinases (MMPs), similar in nature to ADAM10, have been found to cleave within the Aβ domain and consequently modulate amyloidogenesis [138]. These proteinases are found in all central nervous system (CNS) cells and are known to interact with cytokines and chemokines in the regulatory process of neuroinflammation [139]. The MMP family is regarded as Aβ-degrading overall, and some members, such as MMP-9, have been described as cleaving both soluble and fibrillar Aβ [139]. However, while mostly secreted, MMPs have a subfamily of 6 transmembrane proteins known as membrane-type matrix metalloproteinases (MT-MMPs), of which some display pro-amyloidogenic tendencies [138,139]. Of these, MT5-MMP was found in amyloid plaques by immunostaining, and MT1-MMP was identified in microglial cells near plaque agglomerates [139]. A correlation between MT1-MMP increase and a higher abundance of the Aβ precursor fragment, C99, was observed in familial AD (FAD) mice [138]. Additional fragments released during MT5-MMP cleavage, such as a soluble 95 kDa fragment (sAPP95) [140], have also been found to be potentially associated with the progression of AD. Moreover, it was demonstrated that BACE1 inhibition could be partially rescued by MT1-MMP [141], although the exact mechanism is still not understood. Contrary, knockout MT5-MMP mice exhibited a decrease in the Aβ peptide and C99 fragment production at the beginning of the disease, as well as a reduced presence of pro-inflammatory cytokines [140,142].

Another enzyme recently found to be overexpressed in AD and involved in the processing of APP is the type-1 transmembrane protein meprin β [143]. Forms of *N*-terminally truncated Aβ peptides missing the first amino acid, such as Aβ2-40, which did not correspond to the cleavage site of BACE1, were observed in AD and led to the identification of meprin β [143,144]. Membrane-bound meprin β displays β-secretase activity and cleaves APP to release sAPPβ and the pre-amyloidogenic C99 fragment, followed by release of the *N*-terminally truncated Aβ fragments [143]. As with BACE1 cleavage, mutations and PTMs near the meprin β cleavage site can affect the protease’s activity. Phosphorylation at residue Ser750, following APP770 numbering, is shown to increase APP cleavage and consequently leads to increased levels of the highly aggregating Aβ2-40, despite not being located near the β-secretase cleavage site [145]. On the other hand, the A673T mutation was shown to decrease the ratio of Aβ2-40/Aβ1-40 by 70% [143], and the presence of the Swedish mutation prevented the formation of the Aβ2-40 altogether [143]. Interestingly, MT1-MMP appears to mediate the plasma membrane shedding of meprin β [146]. The soluble form of meprin β is released into the extracellular environment, where it results in a cleavage pattern distinct from BACE1 and the release of other truncated nonamyloid forms of APP [147]. A recent study found that knockout of meprin β in mice correlated with a decreased level of Aβ peptides, as well as an improvement in cognitive responses [148]. Another recent discovery is the involvement of the family of cathepsin enzymes in APP processing [149]. Cathepsin B has been found in elevated levels in AD mice models, and its knockdown is correlated to decreased amounts of Aβ isoforms [149]. It is believed that cathepsin B has a unique APP cleavage pattern leading to either increased production of Aβ or clearing of Aβ and maintaining homeostasis [149,150]. Oberstein et al. highlighted the distinct behavior of cathepsin B in different astrocyte organelles, as lysosomal cathepsin B appears to degrade Aβ peptides and non-lysosomal cathepsin B promotes their output [151]. These new findings broaden the scope of the research being conducted on APP processing and its effects on AD progression, as well as additional factors that can affect the process, such as mutations and PTMs.

### 3.2. Role of Mucin-Type O-Glycosylation in APP Processing and AD Pathology

Several reports have indicated that APP is heavily modified with *O*-glycans [112,113,114,115,116]. However, only a few of the 45 threonine and 30 serine residues in the APP sequence have been experimentally identified as glycosylation sites [58,112]. Mass spectrometry studies of the APP695 isoform, produced in CHO cells, revealed the presence of GalNAc and/or core 1 *O*-glycans, including their sialylated versions, at three positions: Thr291, Thr292, and Thr576 [112]. Following this study, a multitude of *O*-glycosylation sites were described for the three different APP isoforms, with only a few variations observed between them, such as an extra glycosylation site at threonine residue (Thr353) on APP770 located within the OX-2 domain [114]. Using APP770 numbering, the consequent sites found on APP that displayed GalNAc and Core 1-like glycans are Thr633, Thr651, Thr652, Ser656, Thr659, Thr663, and Ser667 (Figure 3) [113,114]. Along with these findings, a novel type of tyrosine *O*-glycosylation was discovered to be present on the Tyr681 residue, and a 2.5-times increase in CSF of patients with AD compared to healthy subjects was observed [113]. In 2021, the further exploration of these *O*-glycosites led to the characterization of glycans found on Thr651 and Thr652 as GalNAc, Core 1, and/or its sialylated versions [115]. Most recently, a study presented two novel complex glycosites on Thr269 and Thr274 of APP770, while it also confirmed that most *O*-glycosylation sites are centered near the KPI, OX-2, and Aβ domains [116]. Therefore, the effect of *O*-glycosylation on the processing of APP warrants interest.

APP is glycosylated in the Golgi apparatus prior to its trafficking to the cell membrane [116,152] by the family of enzymes called polypeptide *N*-acetyl-α-galactosaminyltransferases (ppGalNAc-Ts) [153]. The initial transfer of a GalNAc residue to a serine or threonine residue was catalyzed by ppGalNAc-T1, -T2, T3, and -T13, while sialyltransferases were involved in the capping with sialic acid [114,154]. Interestingly, ppGalNAc-T4, -T6, and -T10 were found to be tightly related to the progression of AD [114], and the inhibition of some of these isoforms decreased *O*-glycosylation of APP, leading to a decrease in the production of Aβ peptides [153]. Thus, specificities in residue glycosylation by ppGalNAc-Ts may account for the observed differences in APP processing [114]. Furthermore, the mapping of the *O*-linked glycosylation sites on the APP revealed the presence of several mucin-type *O*-glycosylation sites near the β-secretase cleavage site [115]. Given the distinct effects of mucin-type *O*-glycosylation on the proteolytic activity of various enzymes [155,156], it is reasonable to suggest that glycosylation plays a crucial role in APP processing [62,116,157,158]. Mucin-type *O*-glycans contribute to protein function by regulating proteolytic cleavage, either activating or inactivating target proteins. Additionally, proteolytic cleavage is integral to ectodomain shedding, a process that, in certain cases, is modulated by mucin-type *O*-glycosylation [159]. Additionally, lectin binding assays showed that APP cleaved by α-secretase and β-secretase has different lectin binding patterns [63]. Thus, it is reasonable to assume that APP glycosylation affects its processing [63].

In AD, protein *O*-glycosylation is altered in the CSF, with regions varying between higher or lower expression; however, overall glycosylation levels appear reduced [62,152]. Glycans near the β-secretase cleavage site of APP, such as simple *O*-GalNAc and/or extended and sialylated chains at Thr663, Ser667, or Tyr681, could regulate APP processing by either promoting or inhibiting proteolysis [115,160]. The type of glycan, its site of attachment, neighboring glycosylation, and glycan density are all key factors that can influence APP proteolytic processing. These variables can affect enzymatic accessibility, structural conformations, and overall protein interactions, ultimately shaping APP cleavage and its downstream effects. Singh et al. demonstrated that the presence of GalNAc on Thr663, Ser667, and/or Tyr681 in Swedish-mutated APP peptides influenced their conformation [117]. Glycosylation at Ser667, along with the Swedish mutation, was found to enhance BACE1 activity, whereas modification at Tyr681 reduced BACE1 activity while increasing ADAM10 activity. This shift resulted in partial inhibition of Aβ40 fibril aggregation compared to the native glycosylated peptide [117]. Similarly, Liu et al. highlighted the significance of *O*-glycosylated Tyr681 in limiting Aβ42 aggregation, showing that the presence of GalNAc, Galβ1,3GalNAc, and its sialylated counterpart exhibited inhibitory properties [118]. The role of APP sialylation in AD remains a significant yet underexplored area of research, particularly given its reported impact on protein function [161]. In the CSF of patients with AD, sialyltransferase-mediated sialic acid addition is notably reduced [157]. However, the overexpression of ST6GAL1 has been shown to double the production of Aβ fragments [157], underscoring the critical influence of α-2,6 sialylation in APP processing. Recent findings also reveal that microglia bearing *O*-linked sialylated glycans encircle amyloid plaques, potentially triggering immune responses that target aggregation and fibril formation [162]. The seemingly contradictory effects of sialylation may stem from its dual role in APP processing and immune response modulation. Sialylation can influence proteolytic cleavage, either facilitating or hindering enzymatic access, while also interacting with immune receptors, such as siglecs, to shape neuroinflammatory pathways in AD [114]. This further highlights the critical role of *O*-glycosylation in the processing and clearance of APP, as it can either protect against or promote amyloidogenesis, depending on the type and position of the glycan.

## 4. Regulation of Immune Responses and Neuroinflammation by *O*-Glycan Binding Proteins

The immune system is a crucial player in the defense mechanism processes that protect the body by recognizing and eliminating pathogens and intrusions that threaten its homeostatic constitution. Therefore, in neurodegenerative disorders, such as AD, immune responses occurring in the neuronal space become extremely important. Among the various immune cells involved in immunoregulatory responses, microglia are regarded as the key drivers of neuroinflammation, not only in AD but also in other neurodegenerative diseases [163]. Furthermore, recent research data have demonstrated the importance of glycan structures and their binding partners in the regulation of immune responses and the widespread increase in neuroinflammation [119]. Therefore, by identifying receptors and downstream signaling pathways that promote the secretion of pro-inflammatory cytokines, we can better understand the role of *O*-glycans in these processes and their impact on the progression and pathogenesis of AD.

### 4.1. Immune Homeostasis

Divided into two branches, the innate and adaptive immune systems work seamlessly to employ fighter cells whose purpose is to neutralize foreign pathogens while preserving the body’s own healthy cells. The innate system provides an instant but nonspecific response, making use of barriers (e.g., mucous membranes) and inflammatory pathways (e.g., cytokine release) to combat intrusions [164]. Specifically, dendritic (DC) and natural killer (NK) cells work in tandem with macrophages and other immune cells to jumpstart the destruction of the trespasser [164,165]. The immune response is initiated by employing pattern recognition receptors (PRRs), which detect pathogens with similar conserved structures known as pathogen-associated molecular patterns (PAMPs) or damage-associated molecular patterns (DAMPs), which are secreted by damaged cells [164]. On the other hand, the adaptive system is a slower but highly specific process with long-lasting protection, as it benefits from immunological memory, allowing for faster response if the same intrusion is observed by recognizing it as “non-self” [165]. This branch is mediated by lymphocytes, of which T cells and B cells are some of the main players [165]. Both systems communicate and cooperate through complex signaling pathways to maintain cell homeostasis.

### 4.2. The CNS and the Neuroimmune Signaling Interplay

While the CNS can regulate immune responses via intricate signaling and through activating neuronal pathways that drive the release of either pro- or anti-inflammatory cytokines, the immune system also plays a role in the surveillance of the CNS via circulating immune cells [166]. Interestingly, DCs have been recorded to leave the brain and infiltrate peripheral organs, driving immune responses and leading T cells to the brain to fight off intrusions [166]. In fact, as many as 80% of the immune cells found in the CSF of healthy donors were found to be T cells that have penetrated the CNS through the brain’s layers, which confirms the interplay between the CNS and the immune system [166]. Nonetheless, the CNS still maintains its own specialized immune network, in which the main cells involved are microglia, making up 80% of the immune cells present in the brain [167]. Microglia provide the first line of defense from pathogenic and stress-derived intrusions in the CNS by stimulating inflammatory processes, such as the release of pro-inflammatory cytokines, which are then mitigated by other neuroprotective actions to achieve homeostasis [168]. However, continuous activation of microglial responses driven by poor cellular debris removal can lead to neuroinflammation and eventual neuronal cell death, as is believed to be the case in many neurodegenerative disorders, such as AD [168].

Microglia and the Neuroinflammatory Cascade

The neuroinflammatory cascade is a series of neuronal events triggered by disease or injury in the brain. Initially, astrocytes and resting microglia detect harmful stimuli, such as pathogens or cellular damage, and become activated. Activated microglia can differentiate into two different phenotypes, the pro-inflammatory M1 and anti-inflammatory M2, mirroring those of macrophages outside of the CNS [48]. However, in recent years, there has been discourse regarding the accuracy of these designations, with research suggesting that microglia can adopt various phenotypes, such as ‘dark microglia’, with their role in synaptic functioning and loss [169,170]. Nevertheless, this review will continue to utilize the M1/M2 designation as it provides an accessible point of differentiation for both pathways involved in the onset of disease. In the case of several neurodegenerative disorders, neuronal debris activates microglia to adopt one of the two phenotypes to respond to stimuli. Under moderate disruptions, the M2 configuration leads to the release of anti-inflammatory cytokines (e.g., TGF-β, IL-10, IL-4, and IL-3) and phagocytosis, which work in a dynamic cycle with inflammatory processes that release cytokines around the area of damage to mark it for clearance by other immune cells, therefore maintaining overall homeostasis [171]. However, the persistent presence of foreign intrusions and other activating stimuli prevents the resolution of the inflammatory setting, leading to an overexpression of the M1 phenotype and heightened release of pro-inflammatory cytokines (e.g., TNF-α, IL-6, IL-4, and IL-12) and inhibition of phagocytosis [171]. Another hallmark of neuroinflammation is the disruption of the blood-brain barrier’s (BBB) permeability, allowing the filtration of peripheral immune cells (e.g., T cells, B cells, and NK cells), which can heighten the release of inflammatory cytokines and mediators or directly affect glial processes [172].

Recent studies have suggested that microglia are activated by various Aβ species at different levels [44]. On one hand, fibrillar Aβ triggers phagocytosis via microglia, while oligomers interfere with this process and evoke primarily the release of pro-inflammatory cytokines [44,173]. Further studies expanded on the differences between oligomeric species and demonstrated that small oligomers increased neurotoxicity and microglia activation, as opposed to high molecular weight oligomers [174]. Microglia are also involved in the propagation of the Aβ peptides to non-disease-associated neurons by acting as seeding agents for the formation and spread of new fibrils [175,176], thus confirming the overarching role of these cells in the pathogenesis of AD. Tau has also been shown to activate microglia, while pro-inflammatory cytokines can induce the formation of NFTs by promoting tau phosphorylation [177,178]. Although it’s still unclear what the exact progressive steps are that lead to the aggravated effect neuroinflammation has on AD onset, new research continues to emerge, including the potential role of TREM2 in microglia differentiation and function [179,180]. In addition, the emerging role of *O*-glycans and glycan-binding proteins in the proliferation of neuroinflammation in Alzheimer’s disease and other neurodegenerative disorders is becoming increasingly recognized.

### 4.3. Glycan-Binding Proteins and Immune Response

The complex array of glycans found on the surface of mammalian cells plays key roles in numerous biological processes, including cell-cell communication, migration, and immune recognition [181,182]. Immune cells have evolved to express specialized glycan-binding proteins (GBPs) that interact with and recognize distinct carbohydrate structures displayed on the cell surfaces as antigenic targets [182]. These interactions are essential for mediating various immune responses, such as pathogen recognition and immune cell signaling [182]. Upon glycan binding, GBPs decode the information contained in glycan structures and trigger complex signaling cascades to modulate immune cell activation and differentiation [182].

**Figure 5:**
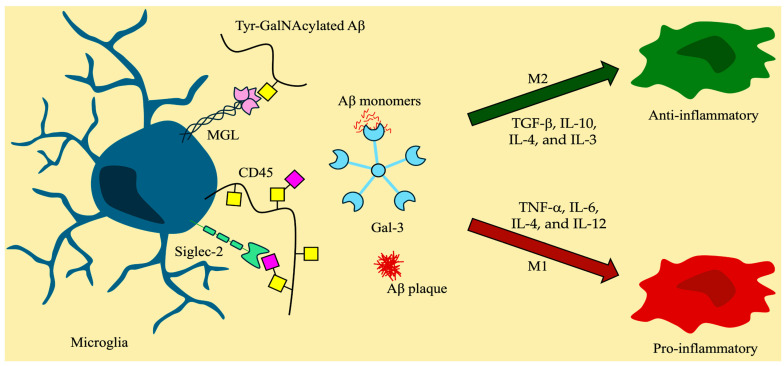
Microglia and the effects of glycan-binding proteins in neuroinflammation. Symbol nomenclature for glycans: a yellow square for *N*-acetylgalactosamine (GalNAc), a yellow circle for galactose (Gal), and a pink diamond for sialic acid.

Another interesting, and underexplored, aspect of *O*-glycans and GBPs is their role in the neuroinflammation cascade and microglia activation (Figure 5). In recent years, the involvement of carbohydrates in the functioning of neuronal synapses and development has become a topic of conversation. Given the knowledge that microglia express several GBPs, which contribute to their activation response [119], opportunities arise to explore the world of *O*-glycans in neuroinflammatory-regulated diseases, such as AD. The effect of *O*-glycosylation on immune activity and neuroinflammation in relation to the three main GBP families—galectins, siglecs, and C-type lectin receptors—will be discussed below.

#### 4.3.1. Galectins and *O*-Glycans

Galectins, first discovered in vertebrates in 1975 [183], are a family of small soluble GBPs involved in the regulation of cell signaling, immune responses, and inflammation [184]. Galectins are distinguished by their specific affinity towards glycans containing β-galactoside moieties in terminal positions of oligosaccharide structures via their ~130 amino acid-long carbohydrate recognition domain (CRD), which can manifest in a single, double, or chimeric form [185,186]. While the N-acetyllactosamine (Galβ1,4GlcNAc) unit is considered the most common ligand across all different types of galectins, each member of the galectin family displays preferences towards a variety of galactosides [187]. Galectins are widely expressed by a variety of cells, with prevalence in immune cells such as activated T and B cells, and macrophages [187]. The interactions between galectins and glycoproteins bearing carbohydrate ligands influence both branches of the immune system by regulating B cell maturation [182], T cell activation [184,187], and overall immune cell differentiation to elicit prompt responses [184,187,188]. While more information appears to be available on the roles of galectin binding to *N*-linked glycans, the interaction of galectins with *O*-glycans, and in particular mucin-type *O*-glycans, has garnered attention [187,189]. Galectin-3 (Gal-3) was found to bind to core 1 mucin-type glycans (TF antigen) and elicit an immunosuppressive response in tumor-expressing environments [190]. Galectin-1 (Gal-1) binding to the glycoprotein CD45 accelerates T-cell apoptosis in a dependent manner when core 2 *O*-glycans are present [191]. On the other hand, Gal-3 binding to CD45 resulted in apoptosis resistance in B-cell lymphoma via CD45 phosphatase activity suppression [188,192]. Isoforms of CD45 were also identified as ligands for Galectin-8 (Gal-8), with one isoform influencing B cell activation and another playing a role in T cell maturation and differentiation, as well as their proliferation [188]. Additionally, galectins are involved in inflammatory responses. For example, Gal-1 has been found to bind to the heavily mucin-type glycosylated CD43 protein on DCs to induce high levels of IL-27 and IL-10 cytokines, known to suppress autoimmune neuroinflammation [188,193]. Therefore, through the diverse roles of galectins as regulators in immune homeostasis, their interactions with glycans are regarded as a point of focus in preventing or delaying disease pathogenesis.

Only galectins 1, 3, 4, 8, and 9 have been reported to be expressed in the CNS and are believed to play a role in the regulation of neurological disorders [194]. The microglial expression of Gal-3 is triggered by inflammatory mediators and promotes their activation in either a pro- or an anti-inflammatory manner, depending on the model [119]. Likewise, Gal-9 is an important player in neuroinflammation by increasing microglial activity [119]. On the other hand, Gal-1 is related to the decrease in microglial activity. Gal-3 has become a key focus in AD due to its strong disease-related expression in microglia [195]. Not only is Gal-3 overexpressed in patients with AD, but it also promotes Aβ oligomerization by binding to Aβ monomers [196]. It was hypothesized that the interaction between the Aβ peptide and Gal-3 is mediated by the presence of core *O*-glycans on the Tyr-10 position in the Aβ1-19 peptide [196]. Interestingly, mice displaying a 40% decrease in Gal-3 expression produced fewer activated microglia and consequent inflammation [196]. Moreover, deletion of Gal-3 in 5xFAD mice resulted in lower microglia-led immune responses and decreased Aβ peptide production [195]. Additionally, Gal-3 was also shown to interact strongly with tau aggregates and promote their propagation to healthy cells [195,197], leading to eventual neuroinflammation. Further research is essential to gain a clearer understanding of how interactions between galectins and *O*-glycans affect the prevalence of neuroinflammation and the progression of AD.

#### 4.3.2. Siglecs and Sialylated *O*-Glycans

Siglecs, or sialic acid-binding immunoglobulin-like lectins, are a family of transmembrane GBPs that specifically recognize terminal sialic acid found in α2,3, α2,6, or α2,8 glycosidic linkages [198]. However, given the sheer level of diversity in complex carbohydrate sialylation, siglecs have evolved to differentiate between distinct glycosidic linkages to elicit specialized immune responses based on the subtle differences between self and non-self-glycoepitope ligands [198,199]. Siglecs are expressed on the surface of almost all human immune cells in strictly localized patterns, such as Siglec-1 on macrophages and Siglec-2 (CD22) on B cells [200], which is why they are considered vital contributors to the regulation of immune cell signaling, activation, and responses [198]. Additionally, the majority of siglecs display immunoreceptor tyrosine-based inhibitory motifs (ITIMs) on their cytoplasmic tails, which are known to suppress transmembrane cell signaling, though some siglecs present activating effects of immune responses [200]. Nonetheless, the specificity of siglecs towards sialylated glycans presents the possibility of truncated mucin-type *O*-glycosylation being a key player in the modulation of immune responses. In fact, CD43 on T cells has been established as a receptor for macrophage-expressed Siglec-1, and their interaction was shown to mediate inflammation in tumor environments [200,201]. Disialylated core 1 *O*-glycans’ expression was found to be increased on naïve T cells, and their presence on different sialomucins, such as CD43, can render them ligands for Siglec-7 and induce T cell signaling pathways to modulate their immune activity [202]. Additionally, the macrophage- and DC-associated Siglec-15 was shown to preferentially bind to sTn, eliciting immune suppression [203]. Moreover, a branched sialylated glycan was recently found to act as a ligand of Siglec-3 (CD33), which is believed to be involved in the pathogenesis of late-onset AD [204], expanding the scope of possibilities for therapeutic interventions in AD.

A limited number of siglecs are known to be expressed in the CNS (e.g., Siglec-1, 3, 4, and 11), and even then, they are only prevalent in disease-associated microglia [194]. The majority of these microglia-expressed siglecs play a role in the progression of neuroinflammatory processes via binding to glycosylated molecules. As an example, the two isoforms of CD33 observed in microglia display opposite effects on the clearance of the Aβ peptide, with the one missing the sialic acid-binding domain (CD33m) promoting Aβ clearance by M1 microglia [205], thus confirming the vital role of sialic acid in AD pathogenesis. Additionally, interactions between CD33 and glycoproteins can hinder the phagocytosis of Aβ accumulations [206]. On the other hand, neuronal Siglec-2 (CD22) ameliorated the effects of neuroinflammation by inhibiting the release of pro-inflammatory cytokine TNFα via binding to the *O*-glycosylated CD45 [119]. Interestingly, soluble CD22 was found to show a correlation with increased tau levels in the CSF [207], while hypersialylation was observed in NFTs [208]. Despite the scarcity of information on the specific interactions between siglecs and *O*-glycans in relation to neuroinflammation, further exploration is crucial to enhance our understanding of disease onset.

#### 4.3.3. C-Type Lectins and *O*-Glycans

C-type lectins (CTLs) are a diverse group of membrane-bound GBPs that display specialized carbohydrate binding distinct to each lectin. Members of the family had been identified for over a century, but it was not until 1993 that a classification of all CTLs was established [209]. CTLs are characterized by a CRD that was described as Ca^2+^-dependent for sugar binding [210]. However, as new research emerged, various CTLs were determined to bind complex ligands in a Ca^2+^-independent fashion, such as Dectin-1 (CLEC7A), which is expressed in DCs [210,211]. CTLs, and specifically C-type lectin receptors (CLRs), are commonly expressed by myeloid cells, such as macrophages [212]. They appear to play an important role in the process by which APCs initiate immune response by relying on intracellular signaling after binding to PAMPs and DAMPs [212]. The binding of the mucin-type *O*-glycan GalNAc by certain CLRs has attracted attention due to its impact on immune response [212,213]. When MGL, expressed on antigen-presenting cells (APCs), binds to CD45 on T cells, it reduces their activation. This results in a decrease in the release and spread of pro-inflammatory cytokines [214]. Therefore, considering that in recent years the neuroinflammatory cascade theory has been proposed to be involved in the progression of most neurodegenerative disorders, the role that CTLs and their specific *O*-glycan binding partners play in this scenario is of great interest.

As previously mentioned, CTLs are found in various cells, including microglia, where they take part in inflammatory responses. The lack of interactions between P-selectin and its mucin-like *O*-glycoprotein ligand (PSGL-1) leads to decreased microglial activation [194]. Interestingly, the binding of the CLR CLEC2 (CLEC1B) to its mucin-type *O*-glycosylated ligand, podoplanin (PDPN), promotes platelet activation [210]. Activated platelets are believed to play a role in increased Aβ production and, thus, neuroinflammation [215]. Studies showed that increased expression of CLEC2 and PDPN is observed in patients with AD, and they showcase a pro-inflammatory effect after injury is experienced [215,216]. Another CTL that has garnered attention for its underexplored role in neuroinflammation and AD is MGL. Human MGL is the only CLR that can specifically bind terminal GalNAc, along with its sialylated complex and is primarily expressed by myeloid APCs like DCs and macrophages, along with brain microglia [55,217]. While the function of MGL in immune responses is mostly regarded as anti-inflammatory, given its involvement in the release of IL-10, its role in neuroinflammation has not been greatly explored [55]. In a study on the autoimmune neuroinflammatory disease multiple sclerosis, MGL was found to be overexpressed on M2 microglia present near inflammation sites, but the relationship between it and the release of IL-10 still needs to be explored [55]. With growing interest in neuroinflammation’s role in triggering AD and the elevated Tn and sTn levels on the cell surface proteins in patients with AD [218], MGL’s specificity towards glycan structure and/or surrounding amino acid epitope is beginning to be explored [219,220,221]. Interestingly, a recent study demonstrated MGL’s affinity for the GalNAc-Tyr moiety, an interaction previously considered unlikely due to the bulkiness and rigidity of the Tyr amino acid [219]. Peptides derived from APP cleavage in CSF bearing the GalNAc-Tyr moiety were also studied and shown to tightly interact with MGL [219]. However, the direct effects that MGL-glycan interactions have on neuroinflammation and AD have not been elucidated, and further research must be conducted to explore their potential as a therapeutic target.

## 5. Conclusions

The intricate processing of APP and its role in AD pathogenesis have been extensively studied, with significant emphasis on its cleavage pathways and the subsequent formation of neurotoxic Aβ peptides. The non-amyloidogenic and amyloidogenic pathways are primarily regulated by α-, β-, and γ-secretases, dictating whether APP processing leads to neuroprotection or the formation of Aβ plaques. In recent years, additional proteases such as MMPs, meprin β, and cathepsins have been identified as contributors to APP processing, expanding the complexity of the regulatory mechanisms involved in AD progression. Moreover, PTMs, particularly mucin-type *O*-glycosylation, have been a critical factor influencing APP cleavage and Aβ production. Specific glycans near enzyme cleavage sites suggest a potential regulatory function in APP processing, either promoting or inhibiting amyloidogenesis. While studies indicate that certain glycosylation patterns may protect against Aβ aggregation, others appear to enhance β-secretase activity, further complicating the understanding of glycosylation’s impact on AD. These findings underscore the need to further study the precise mechanisms by which glycosylation influences APP metabolism. A deeper comprehension of these processes could provide valuable insights into approaches to modulate APP processing and mitigate Aβ accumulation in AD. Importantly, shedding light on the specificity of ppGalNAc enzymes in differential regulations of *O*-glycan attachment and density may clarify the function of these glycans and their contribution to pathogenesis. In addition, the immune system plays a vital role in maintaining homeostasis by recognizing and eliminating harmful pathogens. In the context of neurodegenerative diseases such as AD, immune responses within the neuronal environment are particularly significant. Microglia serve as key regulators of neuroinflammation, contributing to disease progression. Additionally, glycan structures and their interactions have been shown to influence immune responses and neuroinflammation. Elucidating the receptor-ligand interactions, alongside the signaling pathways that drive pro-inflammatory cytokine secretion, can provide valuable insights into the role of *O*-glycans in AD pathogenesis, potentially paving the way for novel therapeutic strategies.

## Data Availability

No new data were created or analyzed in this study.
